# Parasitic Infection from Dogs

**Published:** 1887-12

**Authors:** 


### PARASITIC INFECTION FROM DOGS.

Dr. Ollivier, of Paris, is quoted as follows on this subject by the *British Medical Journal* of November 5, 1887:

"The number of hydatid cysts in a country is in direct proportion to the number of dogs. Facts seem to confirm this theory; for they show that there are more hydatid cysts in hill districts than in the low lands, in the country than in towns. The author cites several cases observed by him, in which the cyst was plainly traceable to previous intercourse with dogs, in patients fond of these animals. A long period, sometimes years, may elapse before the echinococcus develops itself, but the cause is none the less evident. Children are always fond of dogs, and like to play with them, and these pets are affectionate and caressing, and have a bad habit of licking; in this way the animal's tongue may prove a ready means of propagation of the eggs of tænia. It is also possible that they may be inhaled, when, in very dry weather, these eggs are carried about by the wind. This would explain certain cysts in the respiratory organs. The penetration into the human organism, however, generally takes place through the digestive organs by means of salads, green vegetables, fruit and other aliments imperfectly cleaned; or by water from ponds, reservoirs, or wells contaminated by dogs resorting there to drink. The author points out the precautions which should be taken to diminish the danger of propagation of this parasite: greater care in the choice and cleanliness in the preparation of certain articles of food; avoidance of unfiltered water, and particularly of stagnant water of any kind; and, above all, less intimacy with dogs—particularly in the case of children, who should be carefully watched. He condemns altogether house dogs of all kinds, as being a source of continual danger as regards the propagation of the eggs of *tænia echinococcus*."—*Exchange*.

